# Circulating ESM-1 levels are correlated with the presence of coronary artery disease in patients with obstructive sleep apnea

**DOI:** 10.1186/s12931-019-1143-6

**Published:** 2019-08-20

**Authors:** Haili Sun, Fang Fang, Kun Li, Huina Zhang, Ming Zhang, Lichuan Zhang, Juan Li, Yanwen Qin, Yongxiang Wei

**Affiliations:** 1 Department of Otolaryngology, Beijing Anzhen Hospital, Capital Medical University, NO.2 Anzhen Road, Beijing, 100029 China; 2 Key Laboratory of Upper Airway Dysfunction-related Cardiovascular Diseases, Beijing Institute of Heart, Lung and Blood Vessel Diseases, Beijing Anzhen Hospital, No. 2 Anzhen Road, Beijing, 100029 China; 3 Department of Cardiology, Beijing Anzhen Hospital, Capital Medical University, NO.2 Anzhen Road, Beijing, 100029 China

**Keywords:** Obstructive sleep apnea, Endothelial cell specific molecules-1(ESM-1), Coronary artery disease, Biomarker, Endothelial dysfunction

## Abstract

**Background:**

Endothelial dysfunction is one of the most important early indicators of atherosclerosis in obstructive sleep apnea (OSA) patients. Endothelial cell specific molecules-1 (ESM-1), which is a novel endothelial dysfunction marker that may be linked to cardiovascular disease. We investigated to assess whether circulating ESM-1 levels are correlated with the presence of coronary artery disease (CAD) in patients with OSA.

**Methods:**

We performed a cross-sectional study in 228 Chinese OSA subjects, including 185 patients with OSA and 43 controls. The Gensini stenosis scoring system was used to assess the severity of CAD. Circulating ESM-1 levels were measured by Human Magnetic Luminex Screening Assay. The associations between circulating ESM-1 levels and CAD were determined by multivariate logistic regression analysis. The association between ESM-1 levels and Gensini scores was determined by multivariate linear regression analysis.

**Results:**

CAD patients had significantly higher circulating ESM-1 levels compared with non-CAD patients (1279.01[918.52–1770.71] pg/ml vs 585.46[423.61–812.56] pg/ml, *P* < 0.001). After adjusting for confounding factors, we found that circulating ESM-1 levels were an independent risk factor for CAD (OR = 1.633/100 pg ESM-1, 95%CI =1.179–2.262, *P* = 0.003), while circulating ESM-1 levels have no significant correlation with the Gensini score. Furthermore, circulating ESM-1 showed higher discriminatory accuracy in predicting the presence of OSA (AUC:0.910).

**Conclusions:**

Circulating ESM-1 might function as a useful biomarker for monitoring the development and progression of CAD in OSA patients.

**Electronic supplementary material:**

The online version of this article (10.1186/s12931-019-1143-6) contains supplementary material, which is available to authorized users.

## Background

Obstructive sleep apnea (OSA) is a common sleep-related breathing disorder caused by recurrent collapse of the upper airway during sleep leading to intermittent airway obstruction and absence of airflow despite respiratory efforts of the diaphragm against the occluded pharynx. These respiratory disturbances are associated with an increased risk for cardiovascular disease, including coronary artery disease, hypertension, and stokes, atherosclerosis [1–4]. The cardiovascular co-morbidities of OSA are assessed by a variety of techniques including measurement of blood pressure, electrocardiogram, exercise testing, echocardiography, and if needed coronary angiography. However, in contrast to the field of cardiovascular diseases, there is currently no established role for blood biomarkers in the diagnosis and risk stratification of cardiovascular consequences/co-morbidities in patients with OSA [5]. Noninvasive blood biomarkers have increasingly emerged as important alternatives to traditional methods for diagnosing and stratifying the risk of disease at its earliest stage.

ESM-1, previously named endothelial cell-specific molecule-1, is a new biomarker of endothelial dysfunction [6]. Lassalle et al found that ESM-1 was highly expressed in atheromatous plaques, speculating that the increase in secretion of ESM-1 may promote the migration and proliferation of vascular smooth muscle cells and ESM-1 may play a key role in the pathology of atherosclerosis [7]. Previous study have verified that ESM-1 might play a key role in endothelial dysfunction by promoting adhesion between monocytes and endothelial cells in inflammatory disorders [8]. It has been reported that blood levels of ESM-1 are elevated in patients with coronary artery disease (CAD) or OSA [6, 9, 10]. However, there is no study that investigated circulating ESM-1 levels and cardiovascular risk of CAD in OSA patients, the relationship between circulating ESM-1 levels and the development and progression of CAD in patients with OSA has never been fully elucidated. However, the relationship between circulating ESM-1 levels and the development and progression of CAD in patients with OSA has never been fully elucidated. Therefore, the present study aims to investigate the correlation of circulating ESM-1 levels with the presence and severity of CAD in patients with OSA.

## Materials and methods

### Study design

This study was cross-sectional study done in the sleep center (Beijing Anzhen Hospital, China). The study was approved by the ethics committee of Beijing Anzhen Hospital (appoval NO:2017005). All patients gave written informed consent to the ethically approved protocols.

All consecutive patients who underwent overnight full polysomnography study due to snoring, apnea, and excessive daytime sleepiness in the sleep center of Beijing Anzhen Hospital between March 2017 and March 2018 were included in this study. A total of 476 patients were eligible for the study. Subjects younger than 18 years, with central sleep apnea syndrome, heart failure, arrhythmias, stroke, chronic kidney disease, chronic obstructive pulmonary diseases (COPD), pulmonary hypertension, active infections and malignancy were excluded from the study. CAD was defined as present if any of the following characteristics was observed: history of physician-diagnosed CAD, use of medications for CAD; the presence of ≥50% luminal stenosis in at least one major coronary artery in coronary angiography [11]. While if patients have significant cardiac syndromes and not performed coronary angiography before, conventional invasive coronary angiography were performed according to standard protocols by interventional cardiologists blinded to the study protocol. If patients have history of physician-diagnosed CAD or use of medications for CAD were also diagnosed as CAD, no cardiac angiography be performed. All patients gave their written informed consent to participate in the study.

A final total of 228 participants were consecutively enrolled, including 185 patients with OSA and 43 healthy controls (Additional file 1: Figure 1). All the OSA patients were classified into patients with and without CAD. According to the diagnostic standard, the OSA patients were divided into two groups: non-CAD (*n* = 117) and CAD (*n* = 68).

Data on patient characteristics, including clinical/biochemical factors, were collected. Body mass index (BMI) was calculated by dividing weight in kilograms by squared height in meters (kg/m^2^). Blood pressure (BP) was measured at the nondominant arm in the morning of the procedure after a 5 min resting interval. Sleepiness was evaluated using the Epworth sleepiness scale (ESS) [12]. We also recorded the medication status of all the participants (Additional file 2: Table S1).

### Coronary angiography analysis

Conventional invasive coronary angiography was performed according to standard protocols by interventional cardiologists blinded to the study protocol. The Gensini stenosis scoring system was used to assess the severity of CAD [13] by two independent experienced observers. Gensini system grades stenosis of the lumen as 1 for 1–25% stenosis, 2 for 26–50% stenosis, 4 for 51–75% stenosis, 8 for 76–90% stenosis, 16 for 91–99% stenosis and 32 for total occlusion. This score was then multiplied by a factor that accounted for the importance of a lesion’s position in the coronary arterial tree. The multiplication factor for a left main stem lesion was 5. The multiplication factor was 2.5 for proximal left anterior descending artery (LAD) and proximal circumflex artery lesions, 1.5 for a mid-LAD lesion, and 1 for distal LAD, mid/distal circumflex artery, and right coronary artery lesions. The multiplication factor for any other branch was 0.5. The severity of disease was expressed as the sum of the scores for the individual lesions [14].

### Laboratory tests

All blood samples were collected after the participants had fasted overnight. Blood samples were then centrifuged for 10 min at 3000 rpm and 4 °C. Plasma samples were subsequently stored in a freezer at − 80 °C before analysis. Serum triglyceride (TG), total cholesterol (TC), low-density lipoprotein cholesterol (LDL-C), high density lipoprotein cholesterol (HDL-C) levels and fasting blood glucose (FBG), and other routine serum biochemical parameters were measured in a biochemical analyzer (Hitachi-7600, Tokyo, Japan) using blinded quality control specimens in the Department of the Biochemical Laboratory at Beijing Anzhen Hospital.

Magnetic Luminex® Assay is a magnetic bead-based antibody microarray founded upon the sandwich immunoassay principle, which can be used to assess the levels of multiple biomarkers with high sensitivity [15, 16]. Plasm ESM-1 levels were quantitated by using a Human Magnetic Luminex Screening Assay (R&D Systems, Minneapolis, MN, USA). The concentrations of ESM-1 in plasm were determined according to the manufacturer’s instructions [17]. In order to determine the precision of the standards and cytokines levels values obtained by the Luminex platforms, coefficient of variation (CV%) was used to calculate intra-assay performance to determine the precision of results. Intra-assay CV < 10% was acceptable [18]. In our study, the intra-assay CV of standard was < 4.0%. Acquisition was performed on the Bio-Plex system (Bio-Rad Laboratories) with the Bio-Plex 3D reader (Luminex FlexMAP 3D) in combination with xPONENT software version 4.2 (Luminex). Data were analyzed by 5-parameter curve fitting using Bio-Plex Manager software, version 6.1.1 (Bio-Rad).

### Statistical analysis

The study was designed as a cross-sectional study. The sample size was calculated by PASS 11.0 (NCSS, LLC, Kaysville, UT, USA) using logistic regression models, with *P* = 0.9, alpha = 0.05, P0 = 0.5, and odds ratio (OR) = 1.633. The sample size of 228 according to the calculation could satisfy the meet of this study. Continuous variables are expressed as mean ± standard deviation or median (interquartile range) and categorical variables as numerals (percentages). The independent Student’s *t* test for normal distribution and the Wilcoxon rank sum test for asymmetric distribution were used to analyze the differences in continuous variables. The Chi square test was used to analyze categorical variables. The association between circulating ESM-1 and OSA was determined by multivariate logistic regression analysis. The association between circulating ESM-1 levels and other inflammatory factors were also evaluated using multivariable liner regression analysis. To evaluate the predictive power of the identified predictors of CAD, we used receiver operating characteristic (ROC) curves and in particular the associated area under the curve (AUC). The ROC curves were made with maternal factors alone and combined with different factors to find the best performing model. A *P* value of < 0.05 was considered statistically significant. Statistical analysis was performed with SPSS 19.0 (IBM Corp., Armonk, NY, USA).

## Results

### Baseline clinical characteristics of the study population

**Fig. 1:**
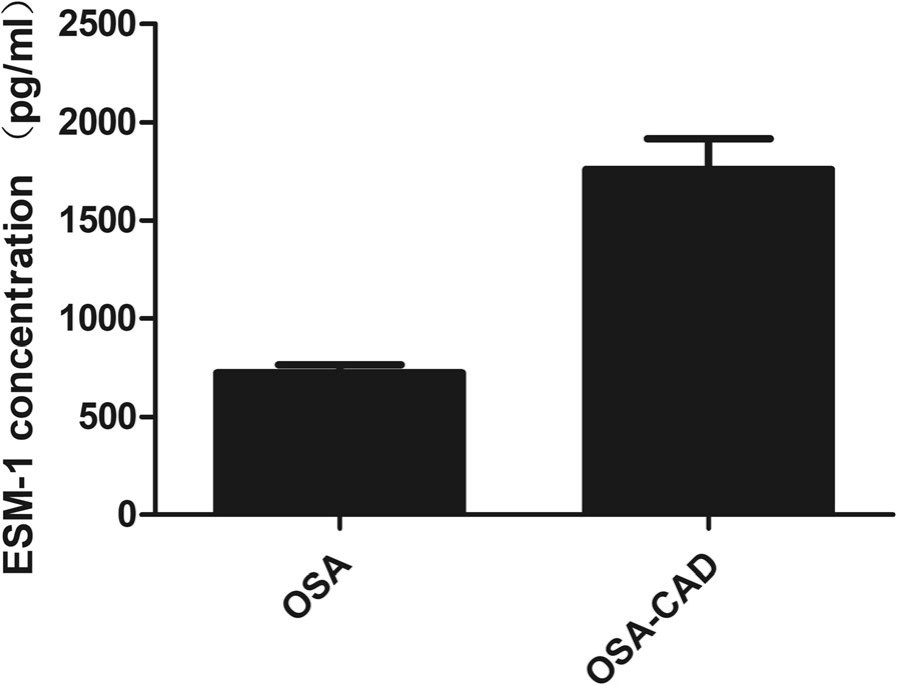
Circulating ESM-1 levels were higher in CAD patients compared with non-CAD patients (1279.01[918.52–1770.71] pg/ml vs 585.46[423.61–812.56] pg/ml, *P* < 0.001)

The baseline clinical characteristics of the study groups are shown in Table 1. The present study included 43 controls, 68 CAD patients and 117 non-CAD patients. There were no differences in BMI (*P* = 0.184), TG (*P* = 0.132), FBG (*P* = 0.088), ALT (*P* = 0.144), AST (*P* = 0.053), SBP (*P* = 0.115) between the two groups. Compared with the non-CAD patients, CAD Patients had a significantly difference in HDL-C levels (*P* = 0.029), ODI(*P* < 0.001), Longest apnea time(*P* < 0.001), CT90%(*P* < 0.001) and arousal index (*P* < 0.001) than those without CAD. As shown in Fig. 1, CAD patients had significantly higher circulating ESM-1 levels compared with non-CAD patients (1279.01[918.52–1770.71] pg/ml vs 585.46[423.61–812.56] pg/ml, *P* < 0.001).

**Table 1:** Baseline clinical characteristics of the study population

Variables	Controls (*n* = 43)	OSA		
		non-CAD (*n* = 117)	CAD (*n* = 68)	*P* value
Male, *n* (%)	29 (67.44%)	97 (82.91%)	61 (89.70%)	0.207
Ages (years)	48.4 ± 16.1	45.6 ± 11.7	56.3 ± 9.9	<0.001^**^
Smoke, *n* (%)	14 (32.56%)	39 (33.30%)	47 (69.12%)	<0.001^**^
Alcohol, *n* (%)	6 (13.93%)	28 (23.93%)	39 (57.35%)	<0.001^**^
BMI (kg/m^2^)	23.77 ± 3.78	27.83 ± 4.51	27.06 ± 3.27	0.184
SBP (mmHg)	120 (110–129)	128 (120–136)	123 (118–132.5)	0.115
DBP (mmHg)	74 (69–80)	81 (74–90)	76.5 (70–89)	0.001^*^
TC (mmol/l)	4.73 ± 1.06	5.09 (4.40–5.95)	4.16 (3.47–4.95)	<0.001^**^
TG (mmol/l)	1.47 (0.99–2.16)	1.54 (1.11–2.10)	1.40 (1.06–1.73)	0.132
HDL-C (mmol/l)	1.23 (1.08–1.50)	1.20 (1.00–1.38)	1.06 (0.87–1.20)	0.029^*^
LDL-C (mmol/l)	2.72 ± 1.08	3.27 ± 0.96	2.53 ± 0.88	<0.001^**^
FBG (mmol/l)	5.13 ± 1.30	5.60 ± 1.63	5.94 ± 1.64	0.088
ALT (mmol/l)	20 (13–27)	24.50 (16–37)	27 (22–37)	0.144
AST (mmol/l)	20.49 ± 4.98	24.39 ± 12.26	27.26 ± 12.42	0.053
Creatinine (mmol/l)	61 (54.1–74.6)	70.65 (60.2–79.05)	67.55 (60.28–79.08)	0.656
Uric acid (mmol/l)	331.30 ± 79.64	408.27 ± 100.13	370.34 ± 90.33	0.012^*^
Homocysteine (umol/l)	9.9 (8.5–15.0)	11.55 (9.10–15.05)	11.8 (9.45–16.10)	0.547
CRP (mg/l)	0.82 (0.27–2.12)	1.58 (0.75–2.97)	1.32 (0.5–3.95)	0.389
ESM-1(pg/ml)	304.24 (114.48–628.32)	585.46 (423.61–812.56)	1279.01 (918.52–1770.71)	< 0.001^**^
AHI	2.80 (1.6–3.70)	37.5 (20.5–60.4)	31.05 (21.88–45.70)	< 0.001^**^
LSaO_2_(%)	92 (91–93)	80 (68.5–86)	85 (75.5–88)	< 0.001^**^
MSaO_2(%)_	97 (95.8–97.07)	96 (95.2–97)	93 (93–95)	0.062
ODI	1.50 (0–2.65)	9.3 (2.38–91.8)	36.1 (26.35–54.25)	< 0.001^**^
Longest apnea time(s)	23.00 (0–39.00)	36.2 (24.5–76.75)	76 (41.5–107)	< 0.001^**^
MAD(s)	24 (18.75–27)	27 (19–35.9)	27 (22.95–31.85)	< 0.001^**^
CT90(%)	0 (0–0.10)	5.8 (0.5–26.5)	14.5 (0.8–34.3)	< 0.001^**^
Arl	10.65 (4.58–15.8)	29.9 (18.3–46.5)	35.6 (22.4–48.5)	< 0.001^**^
ESS	7 (4–10)	12 (8–15)	8 (4–11)	0.003^*^
cIMT (mm)	0.95 ± 0.21	1.17 ± 0.27	1.32 ± 0.34	0.023*

Results are expressed as mean ± standard deviation, median (interquartile range) or n (%). Differences between non-CAD group and CAD group were analyzed by the independent Student t test, χ^2^ text, or Wilcoxon test

*BMI* Body mass index, *SBP* Systolic blood pressure, *DBP* Diastolic blood pressure, *TC* Total cholesterol, *TG* Triglyceride, *HDL-C* High density lipoprotein, *LDL-C* Low density lipoprotein, *FBG* Fasting blood glucose, *ALT* Alanine aminotransferase, *AST* Aspartate aminotransferase, *CRP* C reactive protein, *AHI* Apnea-hypopnea index, *LSaO*_*2*_ Lowest oxygen saturation, *MSaO*_*2*_ Mean oxygen saturation, *ODI* Oxygen desaturation index, *MAD* Mean apnea–hypopnea duration, *CT90* Percentage of cumulative time with oxygen saturation below 90%, *ArI* Arousal index, *ESS* Epworth sleepiness scale

* *P* < 0.05, ** *P* < 0.001

### Association between circulating ESM-1 levels and CAD

We used ordinal logistic regression analysis to estimate associations between CAD and clinical or biochemical variables. As shown in Table 2, older age (OR = 1.091, 95%CI =1.056–1.126, *P* < 0.001), LSaO_2_ (OR = 1.114, 95%CI = 1.065–1.116, *P* < 0.001), and ESM-1 levels (OR = 1.258, 95% CI = 1.150–1.376, *P* < 0.001) were risk factors for CAD. However, HDL-C levels were a protective factor for CAD (OR = 0.900, 95% CI = 0.654–1.239, *P* = 0.520).

**Table 2:** Association between clinical or biochemical variables and CAD

Variables	OR	95%CI	*P* value
Age (year)	1.091	1.056–1.126	< 0.001**
Gender (male = 1, female = 2)	1.906	0.765–4.753	0.166
BMI (kg/m^2^)	0.954	0.884–1.029	0.220
SBP (mmHg)	0.991	0.972–1.014	0.379
DBP (mmHg	0.962	0.934–0.991	0.012*
TC (mmol/l)	0.397	0.278–0.568	< 0.001**
TG (mmol/l)	0.723	0.514–1.016	0.062
HDL-C (mmol/l)	0.900	0.654–1.239	0.520
LDL-C (mmol/l)	0.405	0.273–0.599	< 0.001**
FBG (mmol/l)	1.134	0.942–1.366	0.185
ALT (mmol/l)	1.002	0.988–1.016	0.817
AST (mmol/l)	1.017	0.990–1.045	0.207
AHI (events/h)	0.966	0.951–0.981	< 0.001**
LSaO_2_(%)	1.114	1.065–1.116	< 0.001**
ESM-1(per100pg)	1.258	1.150–1.376	< 0.001**

Dependent variable: CAD

*BMI* Body mass index, *SBP* Systolic blood pressure, *DBP* Diastolic blood pressure, *TC* Total cholesterol, *TG* Triglyceride, *HDL-C* High density lipoprotein, *LDL-C* Low density lipoprotein, *FBG* Fasting blood glucose, *ALT* Alanine aminotransferase, *AST* Aspartate aminotransferase, *AHI* Apnea-hypopnea index, *LSaO*_*2*_ Lowest oxygen saturation, *ESM-1* Endothelial cell specific molecules-1

* *P* < 0.05 in ordinal logistic regression analysis,** *P* < 0.001 in ordinal logistic regression analysis

The association between CAD and circulating ESM-1 levels were tested in different models of logistic regression. Patients with CAD who had higher circulating ESM-1 levels had a higher OR (OR = 1.383/100 pg ESM-1, 95%CI = 1.247–1.534, *P* < 0.001, Table 3). After adjustment for conventional CAD risk factors, including age, sex, BMI, smoking and drinking history, DBP, TG, TC, LDL-C, FBG, ALT and AST, increased ESM-1 levels showed a higher OR of CAD (OR = 1.688/100 pg ESM-1, 95% CI =1.226–2.323, *P* < 0.001). We also adjusted AHI and LSaO_2,_ we found that elevated circulating ESM-1 levels were associated with higher risk of CAD (OR = 1.633/100 pg ESM-1, 95% CI =1.179–2.262, *P* = 0.003).

**Table 3:** Multivariate logistic regression analyses of circulating ESM-1 levels and CAD

	Unadjusted		Model 1		Model 2		Model 3	
	OR (95%CI)	*P*-value	OR (95%CI)	*P*-value	OR (95%CI)	*P*-value	OR (95%CI)	*P*-value
ESM-1 (per 100 pg increase)	1.383 (1.247–1.534)	< 0.001**	1.314 (1.175–1.470)	< 0.001**	1.688 (1.226–2.323)	< 0.001*	1.633 (1.179–2.262)	0.003

Dependent variable: CAD. Model 1: adjusted for age, sex, BMI, smoking, drinking. Model 2: adjusted for Model 1 + DBP, TG, TC, LDL, FBG, ALT and AST. Model 3: adjusted for Model 2 + AHI and LSaO_2_

*BMI* Body mass index, *DBP* Diastolic blood pressure, *TG* Triglycerides, *TC* Total cholesterol, *LDL-C* Low-density lipoprotein cholesterol, *FBG* Fasting blood glucose, *ALT* Alanine aminotransferase, *AST* Aspartate aminotransferase

**P < 0.001 in multivariate analysis, *P < 0.05 in multivariate analysis

### ROC curve analysis for CAD and ESM-1

**Fig. 2:**
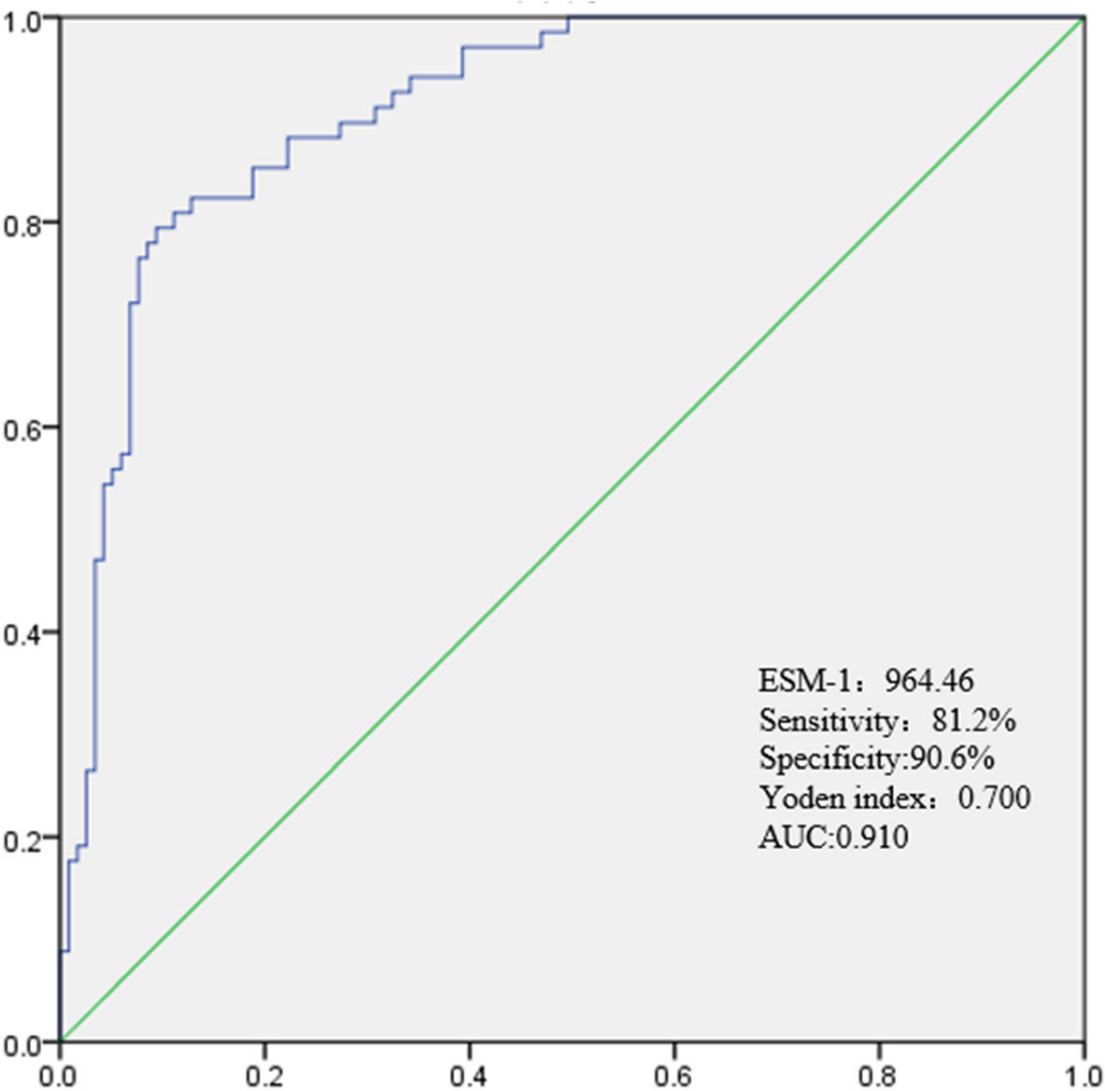
Receiver-operating characteristic (ROC) curve of ESM-1 for predicting CAD. AUC, area under the curve

ROC curve analysis suggested that the optimum ESM-1 level cut-off point for patients with CAD was 964.46 pg/ml, with a corresponding sensitivity and specificity of 81.2 and 90.6%, respectively (AUC = 0.910, 95% CI = 0.846–0.937, *P* < 0.001) (Fig. 2).

### Association between circulating ESM-1 levels and Gensini score

Spearmans’ correlation was used to examine the association of circulating ESM-1 levels and the Gensini score. Circulating ESM-1 levels have no positive correlation with the Gensini score (rho = 0.079, *P* = 0.534, Table 4), which represents the severity of CAD in this study.

**Table 4:** Correlations between Gensini score and ESM-1 level, AHI and LSaO_2_

Variables	rho	*P* value
ESM-1	0.079	0.534
AHI	−0.210	0.295
LSaO_2_	0.064	0.617

*ESM-1* Endothelial cell specific molecules-1; *AHI* Apnea-hypopnea index, *LSaO*_*2*_ Lowest oxygen saturation

*P < 0.05 in Spearmans’ correlation analysis, ** *P* < 0.001 in Spearmans’ correlation analysis

## Discussion

In this study, we found that circulating ESM-1 levels of CAD patients with OSA were significantly elevated compared with non-CAD patients. Circulating ESM-1 levels were positively associated with the severity of CAD after adjusting for confounding factors. Circulating ESM-1 levels were an independent risk factor for CAD. To the best of our knowledge, the present study is the first addressing the relationship between ESM-1 levels and CAD in OSA patients. ESM-1is a marker for vascular pathology and a mediator of inflammation and adhesion, strongly associated with cardiovascular disease [19]. It is secreted upon stimulation by cytokines, namely tumor necrosis factor-α (TNF-α), interleukin (IL)-1 and microbial lipopolysaccharide, as well as by proangiogenic factors such as vascular endothelial growth factor (VEGF) [20]. Via its interaction with intercellular adhesion molecules, ESM-1 exhibits a well-described inhibitory role on leukocyte binding to the vascular endothelium. These properties have highlighted its potential role as a biomarker of endothelial dysfunction and inflammation. In conclusion, since ESM-1 release probably reflects vascular damage and risk factors, its measurement may provide useful information for the management of patients with OSA.

Endothelial dysfunction is considered as the initial lesion in the progress of early atherosclerosis [21]. OSA is one of the most important factors in the pathogenesis of CAD, ESM-1 may play an important role in regulating cell adhesion and raised circulating levels may reflect endothelial dysfunction. Tadzic [22] et al confirmed that the decrease of ESM-1 could reduce the activation of endothelial cells, thus delaying the progress of atherosclerosis. Kose et al. demonstrated that plasma ESM-1 concentrations correlated positively with both markers of inflammation, and ESM-1 levels were significantly increasing in patients with acute coronary syndrome (ACS) compared with the control group. There was no relationship between the Gensini and SYNTAX score and ESM-1 levels; however, there was a significant relationship between ESM-1 levels and the number of diseased epicardial vessels, predicting circulating ESM-1 were associated with the burden of CAD instead of severity of CAD [19].

OSA is associated with an increased risk of cardiovascular disease. Endothelial dysfunction is widely regarded as being involved in the development of atherosclerosis. Carotid artery intima–media thickness (cIMT) is a marker of subclinical atherosclerosis [23]. This result is in accordance with previous studies by Wang et al and Balta et al which revealed that serum ESM-1 levels correlated positively with cIMT in patients with hypertension and psoriasis [24, 25]. Gensini stenosis score is a valid and reliable scoring system that assesses the extent severity of CAD [14]. One of the important findings of the present study was that there was no significant correlation between serum ESM-1 levels, Gensini and SYNTAX score [24]. Kanbay A et al found that ESM-1 levels were significantly higher and flow-mediated dilatation (FMD) measurements were lower in patients with OSA compared to healthy controls and observed a strong negative correlation between serum ESM-1 level and FMD [6]. In our study, we also discuss the relationship of cIMT between these two groups, and found that cIMT were lower in patients with OSA compared to CAD groups. ESM-1 may be a surrogate endothelial dysfunction marker and may have a functional role in endothelium-dependent pathological disorders. All these results suggested that elevated circulating ESM-1 levels might be also correlated with the progression of CAD in OSA patients [19, 25].

Our study showed that circulating ESM-1 levels were positively correlated with age, BMI, SBP, DBP (Additional file 3: Table S2), which can be explained that our study is a cross-sectional study, CAD often occurs when OSA progresses to a certain extent. Epidemiological evidence indicates that blood lipid levels increase with increasing age [26]. However, in our study consistent lower LDL and TC in CAD patients may be the effect of statins or lipid lowering drugs, we found no significant difference in the expression levels of ESM-1 between patients with oral lipid-lowering drugs and those without oral lipid-lowering drugs. In this study, we found that there were no differences in the levels of circulating ESM-1 between the two genders. There is a higher prevalence of smoking and drinking in patients with CAD and OSA. In order to avoid a confounding effect, we also adjusted for sex, smoking and drinking in this study. A previous study showed that BMI are positively associated with ESM-1 levels [6]. While there were no differences in the levels of ALT and AST in CAD compared with non-CAD in our study. In order to avoid a confounding effect, we also adjusted for BMI, ALT and AST in this study. Hypertension, diabetes and renal function is independently associated with circulating ESM-1 levels and ESM-1 levels are closely correlated with blood pressure, FBG, serum uric acid and creatinine levels [26–28]. We adjusted for blood pressure, FBG, serum uric acid and creatinine levels in this study.

Care was taken to avoid bias in our study. Human Magnetic Luminex Screening Assay was performed according to the manufacturer’s instructions by a trained experimenter who was unaware of patients’ clinical data. Moreover, in statistical analysis, adjustments were made for the confounding effects of risk factors for CAD and circulating ESM-1 levels. Finally, propensity score matching was used to reduce the effects of outcome-selection bias. This study has some limitations. First, it was a cross-sectional study, which indicated that it could only show associations, not causality. Second, some of the patients with OSA and some controls were taking drugs, which may affect circulating ESM-1 levels in this study. Third, all of the participants were Chinese, the findings may not be generalizable to other ethnicities. Our findings should be confirmed in other populations.

ESM-1 is a candidate to become a reliable inflammatory indicator of endothelial dysfunction developing before the presentation of the apparent cardiovascular diseases. Therefore, ESM-1 has the potential to shape the therapies regarding the primary prevention of the cardiovascular diseases. Further studies are needed to verify this propose.

## Conclusions

In summary, we revealed that circulating ESM-1 levels were independently correlated with the presence of CAD in patients with OSA. These findings indicated that ESM-1 might function as a useful biomarker for monitoring the development and progression of CAD in OSA patients. The efficacy of therapies to target ESM-1 to delay the degenerative process of CAD warrants further investigations.

## Additional files

Additional file 1: **Figure S1.** Flow chart of inclusion cases and controls in this study. (TIF 463 kb)

Additional file 2: **Table S1.** List of medications for participants. (DOCX 15 kb)

Additional file 3: **Table S2.** Correlations between clinical variables and circulating ESM-1 levels. (DOCX 16 kb)

## Data Availability

All the data from this manuscript is publically available.

## Abbreviations

CAD: Coronary artery disease;
ESM-1: Endothelial cell specific molecules-1
OSA: Obstructive sleep apnea
