# Supplementary figures and images for: Circulating ESM-1 levels are correlated with the presence of coronary artery disease in patients with obstructive sleep apnea
Source: Respir Res. 2019 Aug 20;20:188. doi: 10.1186/s12931-019-1143-6 (PMC6701084; doi:10.1186/s12931-019-1143-6)

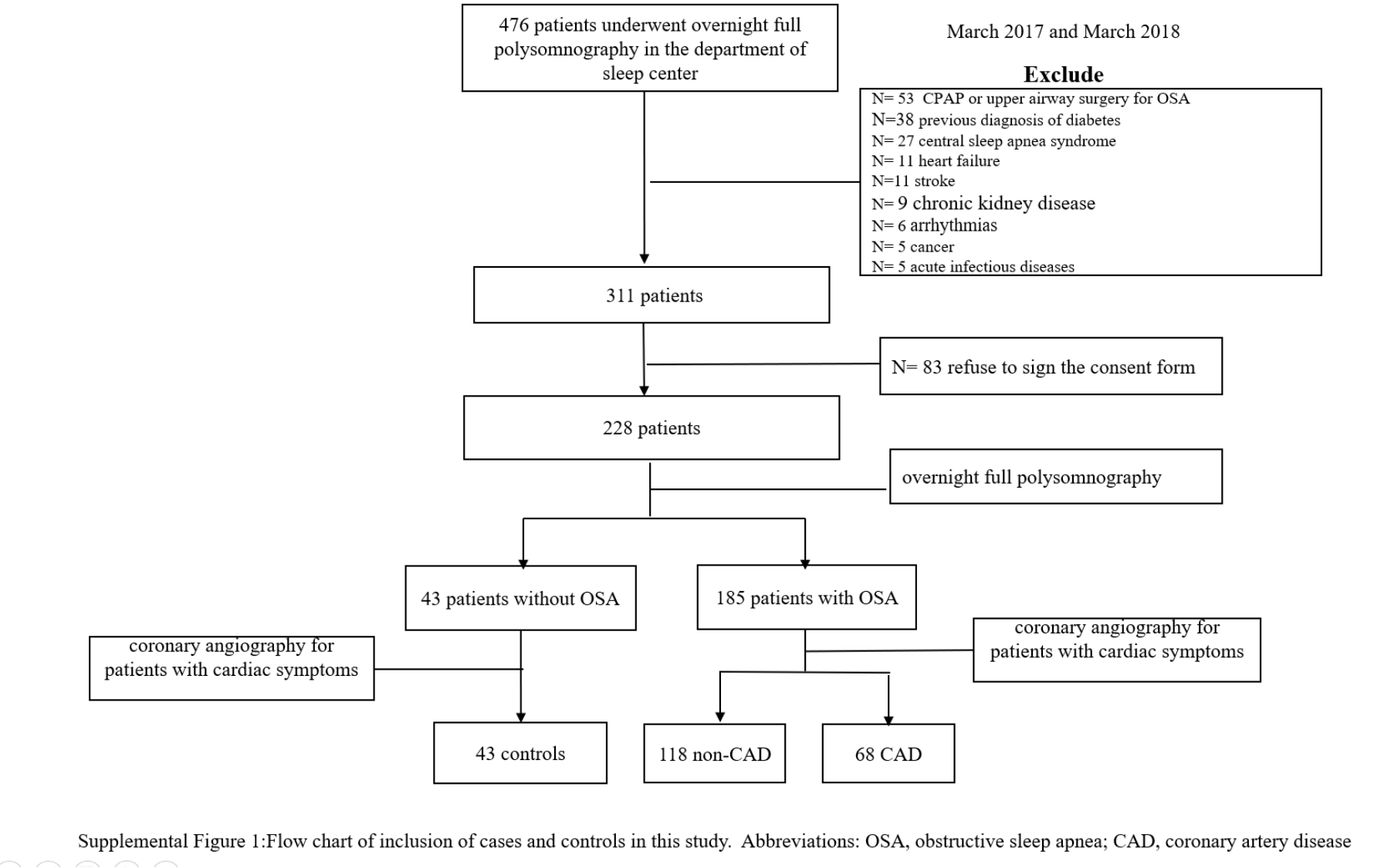

Supplement: Supplementary file 1 — Figure S1. Flow chart of inclusion cases and controls in this study. (TIF 463 kb) [file 12931_2019_1143_MOESM1_ESM.tif]
