# Supplementary material for: Circulating ESM-1 levels are correlated with the presence of coronary artery disease in patients with obstructive sleep apnea
Source: Respir Res. 2019 Aug 20;20:188. doi: 10.1186/s12931-019-1143-6 (PMC6701084; doi:10.1186/s12931-019-1143-6)
Supplement: Supplementary file 2 — Table S1. List of medications for participants. (DOCX 15 kb) [file 12931_2019_1143_MOESM2_ESM.docx]

| **medication** | **non-CAD** | **CAD** | **Control** |
| --- | --- | --- | --- |
| Statins | 19 | 61 | 4 |
| Ezetimibe | 12 | 20 | 0 |
| β-blocker | 15 | 38 | 1 |
| Aspirin | 9 | 61 | 0 |
| Clopidogrel | 11 | 59 | 1 |
| Nitrates | 2 | 38 | 0 |
| Others | 7 | 35 | 2 |

**Supplemental Table S1. List of medications for participants**
