# Supplementary material for: Circulating ESM-1 levels are correlated with the presence of coronary artery disease in patients with obstructive sleep apnea
Source: Respir Res. 2019 Aug 20;20:188. doi: 10.1186/s12931-019-1143-6 (PMC6701084; doi:10.1186/s12931-019-1143-6)
Supplement: Supplementary file 3 — Table S2. Correlations between clinical variables and circulating ESM-1 levels. (DOCX 16 kb) [file 12931_2019_1143_MOESM3_ESM.docx]

**Table S2.** **Correlations between** **clinical variables and circulating ESM-1 levels**

|  | All subjects (n=228) | | |
| --- | --- | --- | --- |
| Variables | rho | P-value | |
| Age (years) | 0.409 | <0.001** | |
| BMI (kg/m^2^) | -0.255 | <0.001** | |
| SBP (mmHg) | -0.314 | <0.001** | |
| DBP(mmHg) | -0.352 | <0.001** | |
| FBG (mmol/L) | -0.225 | 0.001 | |
| TG (mmol/L) | -0.019 | 0.780 | |
| TC (mmol/L) | -0.288 | <0.001** | |
| LDL-C (mmol/L) | -0.010 | 0.879 | |
| HDL-C (mmol/L) | 0.169 | 0.013 | |
| UA (umol/L) | -0.152 | 0.025 | |
| CR (umol/L) | -0.242 | 0.001 | |
| ALT (U/L) | 0.134 | 0.059 | |
| AST (U/L) | -0.164 | 0.022 | |
| γ-GT (U/L) | -0.276 | <0.001** | |
| LSaO_2_ | 0.525 | <0.001** | |
|  |  |  |  |

Abbreviations: BMI, body mass index; SBP, systolic blood pressure; DBP, diastolic blood pressure; FPG, fasting plasma glucose; TG, triglycerides; TC, total cholesterol; LDL-C, low-density lipoprotein cholesterol; HDL-C, high-density lipoprotein cholesterol; UA, uric acid; CR, creatinine; AST, aspartate aminotransferase; ALT, alanine aminotransferase; γ-GT, γ-glutamyltransferase; LSaO_2_, lowest oxygen saturation.*P<0.05, **P<0.001.
